# Capturing asymmetry in COVID-19 counts using an improved skewness measure for time series data

**DOI:** 10.1016/j.mex.2023.102353

**Published:** 2023-09-04

**Authors:** Sudeep R. Bapat

**Affiliations:** Indian Institute of Management, Indore, India

**Keywords:** Skewness, Time series, ARIMA, Non-stationarity, COVID-19, A new skewness measure for time series data, MSC:, 37M10, 62M10, 91B84

## Abstract

Capturing asymmetry among time series is an important area of research as it provides a range of information regarding the behaviour and distribution of the underlying series, which in turn proves to be useful for prediction. Classically, this can be achieved by modeling the skewness of the underlying series, usually using the standard measure. We present here an improved measure of skewness for time series which are integrated by a certain order, which is easy to calculate and proves to be advantageous over the existing one. We complement our methodology by implementing it to represent the heavy asymmetry among the daily COVID-19 case counts of several countries.

• Improved skewness measure proves to be better than the usual skewness measure for time series data
• This new measure is applied on COVID-19 daily counts to capture the asymmetry appropriately

## Graphical abstract

**Figure:**
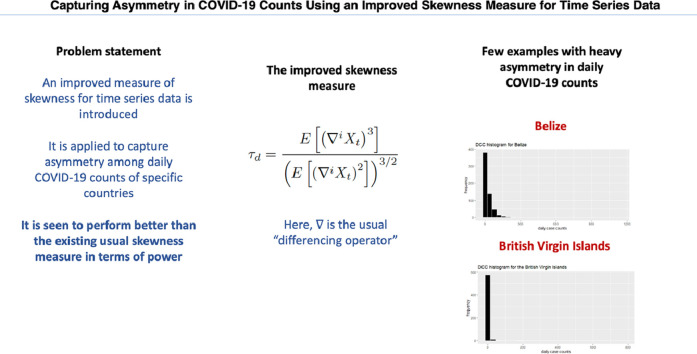

Specifications table

**Table:** 

Subject area:	Mathematics and Statistics
More specific subject area:	Time Series
Name of your method:	A new skewness measure for time series data
Name and reference of original method:	NA
Resource availability:	NA

**Method details**

• In this article, a new measure to capture asymmetry among time series is introduced. Rather than applying the usual existing skewness measure, this new measure can be applied to series which are integrated with a certain order
• This new measure proves to be better than the usual skewness measure in terms of its power, based on different illustrations

## Introduction

Since the outbreak of the novel coronavirus (COVID-19), there has been abundant literature focusing on analyzing the different perspectives and features of the virus. The impact of this global pandemic is so huge that till date there have been over 210 million cases recorded as per the WHO. There is thus an ever growing need of such analyses, to monitor the path of the pandemic. Moreover, numerous statistical approaches have been documented in this regard which are capable of predicting or forecasting the case totals through an underlying model. To list a few, [7] proposed a statistical model to analyze the virus spread specifically in Spain and Italy. Sarkar et al. [10] adopted a similar approach to study the virus spread in India. Arora et al. [3] designed a deep learning approach to predict the number of positive cases, whereas [12] proposed logistic and machine learning models for prediction. A general idea about the awareness of this issue among people can be gauged via the number of “country specific” analyses undertaken by many authors. Just to name a couple, [11] introduced a stochastic modeling approach to analyze the virus prevalence in East African countries or [9] analyzed the predictive modeling of confirmed cases in Nigeria.

Among all these, a specific aspect of analysis is the amount of symmetry or asymmetry present in the daily case numbers. Of course this amounts to analyzing the impact of skewness in such data. A largely asymmetric data distribution clearly poses multiple problems in estimation, be it point estimation or intervals. Typically a 95% interval for the average daily counts in such situations would present a false picture about the true coverage level. Further, obtaining an idea about the skewness also indicates the direction of deviations from the mean. The skewness measure finds its use predominantly in the finance literature, say to describe the returns of a stock. However there have been some attempts made to connect this idea to the current COVID-19 modeling. Particularly, a good reference paper is by Akhtar [2], where the pandemic curves are analyzed through a probability density function with associated skewness and kurtosis measures. Our focus in this paper will be to adopt a time series approach to model the number of daily cases, and propose a new measure of skewness which proves to be better than the classical one. In literature there have been several papers introducing new measures of skewness but clearly, all these may not be easily applicable to a time series data. A few references in this regard are that of [6] who proposed a robust measure of skewness or [1], who presented a skewness measure termed as the “split sample skewness”. From a time series perspective, a paper by Bai and Ng [4] aims to present the sampling distributions for the coefficient of skewness and kurtosis along with a joint test of normality for time series observations.

Following is an outline of this paper: Section ‘A proposed measure of skewness’ presents the proposed measure of skewness along with a few interesting properties for the same. Section ‘Simulations and comparisons’ gives a simulation analysis by adopting a certain bootstrap methodology to compare the powers of the discussed skewness measures, whereas we present a real data example pertaining to modeling the daily covid numbers for a batch of selected countries in Section ‘Real data analysis’. Brief conclusions are included in the 5th Section.

## A proposed measure of skewness

Let ${\left\{X_{t}\right\}}_{t=1}^{T}$ be a time series with mean μ and standard deviation σ. Further let $\mu_{r}=E\left[{\left(x-\mu \right)}^{r}\right]$ be the rth central moment of $X_{t}$ with $\mu_{2}=\sigma^{2}$. The classical measure of skewness (denoted by γ hereafter) which has been implemented even to time series is given by,

(1) $$\gamma =\frac{\mu_{3}}{\sigma^{3}}=\frac{E[{\left(x-\mu \right)}^{3}]}{E{\left[{\left(x-\mu \right)}^{2}\right]}^{3/2}}.$$

We now propose a new measure of skewness, where the focus is more on time series which are integrated with a certain order (say d). In other words, series which are non-stationary and have a certain trend component. The idea behind its design stems from Erdem et al. [8] and Bapat [5]. We denote the new measure by $\tau_{d}$, which is defined as,

(2) $$\tau_{d}=\frac{E\left[{\left(\nabla^{d}X_{t}\right)}^{3}\right]}{{\left(E\left[{\left(\nabla^{d}X_{t}\right)}^{2}\right]\right)}^{3/2}},$$

where, ∇ is the usual differencing operator and in general is given by,

(3) ∇dXt=∑i=0d(di)(−1)iXt−i.

Here, d=0,1,2,… will depend on the order of integration of the series. Specifically, just as an illustration, $\nabla X_{t}=X_{t}-X_{t-1}$ and $\nabla^{2}X_{t}=X_{t}-2X_{t-1}+X_{t-2}$ and so on. Ideally, to capture majority of the real world series a reasonable degree of trend is either 0,1,2 or 3 which represents stationary, linear, quadratic and cubic trends. Our priority in this paper will be to elaborate on the skewness measures $\tau_{1}$ and $\tau_{2}$. In the next section, we will present a variety of integrated time series models by considering different distributions for the innovations. Now again for convenience and completeness, following are specific skewness measures pertaining to different orders of integration d, and are given by:

(4) $$\tau_{1}=\frac{E\left[{\left(X_{t}-X_{t-1}\right)}^{3}\right]}{{\left(E\left[{\left(X_{t}-X_{t-1}\right)}^{2}\right]\right)}^{3/2}}$$

and,

(5) $$\tau_{2}=\frac{E\left[{\left(X_{t}-2X_{t-1}+X_{t-2}\right)}^{3}\right]}{{\left(E\left[{\left(X_{t}-2X_{t-1}+X_{t-2}\right)}^{2}\right]\right)}^{3/2}}$$

The following subsection provides a few Lemmas associated with the newly proposed measure of skewness $\tau_{d}$. For brevity alone, we will restrict ourselves to $\tau_{1}$. One can easily extend these for higher values of d.

Lemma 2.1 *If*${\left\{X_{t}\right\}}_{t=1}^{T}$*represents a symmetrically distributed time series, then*$\tau_{1}=0$*.*

Proof Now without loss of generality we can assume that $X_{t}$ is symmetric around 0. This means that $X_{t}$ and $-X_{t}$ will have the same distribution. Similarly, $X_{t-1}$ and $-X_{t-1}$ also have the same distribution. Hence clearly, $X_{t}-X_{t-1}$ and $-(X_{t}-X_{t-1})$ also have an identical distribution. Therefore,

(6) $$\begin{array}{cc} E\left[{\left(X_{t}-X_{t-1}\right)}^{3}\right] & =E\left[{\left\{-\left(X_{t}-X_{t-1}\right)\right\}}^{3}\right]=E\left[{\left(-1\right)}^{3}{\left(X_{t}-X_{t-1}\right)}^{3}\right]\\ & =E\left[-{\left(X_{t}-X_{t-1}\right)}^{3}\right]=-E\left[{\left(X_{t}-X_{t-1}\right)}^{3}\right]. \end{array}$$

Hence, $E\left[{\left(X_{t}-X_{t-1}\right)}^{3}\right]$ and analogously $\tau_{1}$ equals 0. □

The following Lemmas outline properties of $\tau_{1}$ under non-stationarity and cointegration respectively.

Lemma 2.2 *Let*${\left\{X_{t}\right\}}_{t=1}^{T}$*represent a first-order random walk given by:*

(7) $$X_{t}=X_{t-1}+e_{t},$$

*where*$e_{t}$*is a Gaussian white noise sequence with mean 0 and variance 1. Then,*$\tau_{1}=0$*.*

Proof If we simulate a large number of $X_{t}$ sequences, we clearly have:

(8) $$E\left[{\left(X_{t}-X_{t-1}\right)}^{3}\right]=E\left(e_{t}^{3}\right)=0.$$

And hence, $\tau_{1}=0$. □

Lemma 2.3 *Let*${\left\{X_{t}\right\}}_{t=1}^{T}$*and*${\left\{Y_{t}\right\}}_{t=1}^{T}$*be two cointegrated processes given by,*

$$X_{t}=X_{t-1}+u_{t}$$

$$Y_{t}=aX_{t}+v_{t},$$

*where*$u_{t}$*and*$v_{t}$*are independent Gaussian white noise sequences with means 0 and variances 1 and*a*is a constant. Then, skewness of*$Y_{t}=0$*.*

Proof Now according to the above constructions,

$$\begin{array}{cc} & E\left[{\left(Y_{t}-Y_{t-1}\right)}^{3}\right]=E\left[{\left\{\left(aX_{t}+v_{t}\right)-\left(aX_{t-1}+v_{t-1}\right)\right\}}^{3}\right]\\ & =E\left[{\left\{a\left(X_{t}-X_{t-1}\right)+\left(v_{t}-v_{t-1}\right)\right\}}^{3}\right]=E\left[{\left(au_{t}+v_{t}-v_{t-1}\right)}^{3}\right]=0 \end{array}$$

 □

## Simulations and comparisons

We will now present results for a number of different time series processes, with varying orders of integration. Towards this, a specific bootstrap test for the skewness measure will be established. This approach will be along the lines of that given by Adil et al. [9]. Now since we are dealing with time series, a usual way to introduce a symmetry or asymmetry among the series is to fix distributions for the innovation terms in each case. Specifically, for inducing symmetry we consider N(0,1) and $t_{5}$ distributions. Whereas, to induce asymmetry in the series, we consider $\chi_{5}^{2}$ and Lognormal (LN) distributions. Further, we consider several ARIMA models integrated with orders of either 1 or 2, along with the above mentioned innovation distributions. We fix the AR parameters as 0.7 and 0.2 in each case. Table 1 outlines the time series processes considered for analysis.

**Table 1:** Processes under consideration.

	Model	Innovation distribution
1	ARIMA(2,1,0)	$t_{5}$
2	ARIMA(2,1,0)	N(0,1)
3	ARIMA(2,2,0)	$t_{5}$
4	ARIMA(2,2,0)	N(0,1)
5	ARIMA(2,1,0)	$\chi_{5}^{2}$
6	ARIMA(2,1,0)	LN(0,1)

Clearly, series 1,2,3 and 4 represent a symmetric series whereas 5 an 6 are asymmetric. The next subsection outlines the said bootstrap test along with a power comparison methodology.

### Bootstrap test and power comparisons

As seen in the previous section, our newly proposed skewness measure also takes a value 0 when the underlying series is symmetric. We hence design an appropriate bootstrap test for skewness, which happens to be robust towards the choice of critical values and the initial sample. Without loss of generality, we assume that a series ${\left\{X_{t}\right\}}_{t=1}^{T}$ is symmetric around m, where m is the general central parameter (either mean or median). The null hypothesis under consideration and pertaining to symmetry can be expressed as,

(9) $$H_{0}:F\left(x+m\right)-F\left(m\right)=F\left(m\right)-F\left(m-x\right),$$

where F stands for the distribution of the series $X_{t}$, and x denotes a generalization of $X_{t}$. Now, a suitable bootstrap approach involves the following steps:

Step 1 Let $T^{*}$ be an appropriate test statistic which measures skewness. The null hypothesis will be rejected if $T^{*}$ is significantly different from 0.
Step 2 Construct 2T linear transformations of $X_{t}$ which give rise to transformed series ${\left\{Y_{t}\right\}}_{t=1}^{T}$ and ${\left\{Z_{t}\right\}}_{t=1}^{T}$ as follows: $y_{1}=x_{1}-m,y_{2}=x_{2}-m,\ldots ,y_{T}=x_{T}-m$ and $z_{1}=m-x_{1},z_{2}=m-x_{2},\ldots ,z_{T}=m-x_{T}$. Finally let C be a collective sample of the transformed series. This way, the initial series is symmetrized around m.
Step 3 Generate a bootstrapped series $B^{*}=\left\{B_{1},B_{2},\ldots ,B_{T}\right\}$, where the elements are drawn from C. Then find out the value of the test statistic $T^{*}\left(B^{*}\right)$. Repeat the bootstrap sampling say 10,000 times and get a collection of corresponding $T^{*}$ values.
Step 4 Order the test statistic values $\left(T_{1}^{*}<T_{2}^{*}<\ldots <T_{10,000}^{*}\right)$ and find out the upper and lower 2.5% thresholds for this collection of $T^{*}$ values. Denote them by $T_{25}^{*}$ and $T^{*}975$ respectively. If the observed test statistic falls in the 95% interval, the hypothesis of symmetry will be accepted or otherwise rejected.

Now, in order to compare the powers of the existing skewness measure (γ) with the newly proposed measures $\left(\tau_{1},\tau_{2}\right)$ we adopt the following strategy:

• Apply the bootstrap algorithm outlined above, to get the required sorted test statistic values along with the quantiles.
• Simulate the underlying series along with the corresponding error distribution a fixed number of times (say 10,000).
• If the error distribution is assumed to be symmetric, count the percentage of correct acceptances of the null hypothesis, which will be the power of the test. Similarly, if the error distribution is assumed to be asymmetric, count the percentage of correct rejections of the null hypotheses, which will be the power of the test in that case.

**Fig. 1:**
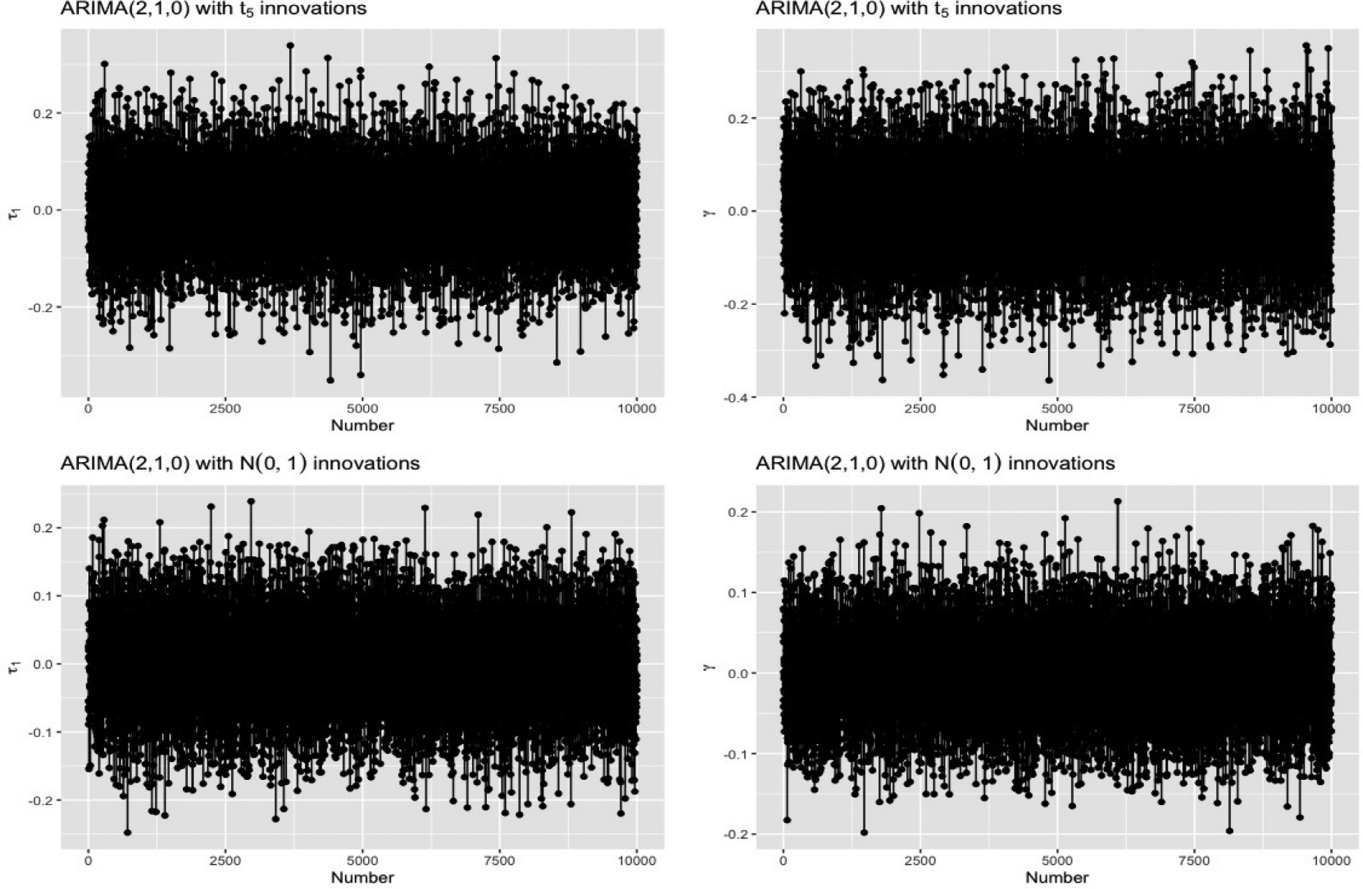
Estimates for the usual skewness measure (left) versus the new skewness measure (right).

We apply the above bootstrap algorithm along with the power comparison to all the models listed in Table 1. These analyses are outlined in Table 2. We also report the means of the calculated skewness measures $\left(\bar{\gamma },\left\{{\bar{\tau }}_{1},{\bar{\tau }}_{2}\right\}\right)$ obtained from the 10,000 simulated series. Ideally, if a series is assumed to be symmetric, the average estimated skewness measure should be close to 0 and if a series is assumed to be asymmetric, it should be away from 0. The powers of the three measures are denoted by $P_{1},P_{2}$ and $P_{3}$ respectively. Clearly, we will only compare $\bar{\gamma }$ and ${\bar{\tau }}_{1}$ if the order of integration is 1, and all three measures if the order of integration is 2. For illustration purposes, Fig. 1 contains side-by-side plots of the obtained estimates under the usual and the newly proposed skewness measures, for the ARIMA(2,1,0) model under $t_{5}$ and N(0,1) innovations. As seen from the figure, the estimates are much tighter around 0, for the newly proposed skewness measure which suggests that it represents a symmetric distribution more adequately.

**Table 2:** Power comparisons for the underlying series.

Model	Innovation distribution	$\bar{\gamma }$	$P_{1}$	${\bar{\tau }}_{1}$	$P_{2}$	${\bar{\tau }}_{2}$	$P_{3}$
ARIMA(2,1,0)	$t_{5}$	0.0003	0.1175	0.0002	0.1786	–	–
ARIMA(2,1,0)	N(0,1)	−0.009	0.1114	0.002	0.1629	–	–
ARIMA(2,2,0)	$t_{5}$	0.0028	0.0101	0.0037	0.0753	0.0008	0.0979
ARIMA(2,2,0)	N(0,1)	0.0034	0.0117	0.0006	0.0714	0.0002	0.1147
ARIMA(2,1,0)	$\chi_{5}^{2}$	0.01	0.2515	1.026	0.9016	–	–
ARIMA(2,1,0)	LN(0,1)	0.05	0.2934	1.127	0.9528	–	–

Now as an assistance, we provide a point wise description of the entire methodology, which will help anyone to navigate through the process smoothly. We adopt the following steps to find and compare the new skewness measure with the usual ones.

• Assuming a time series ${\left\{X_{t}\right\}}_{t=1}^{T}$ is non-stationary (integrated with an order d), we take the dth difference of $X_{t}$ and find the new measure of skewness $\left(\tau_{d}\right)$ given in Eq. (2).
• We also find the usual measure of skewness (γ) given in Eq. (1).
• We repeat the above steps for simulated series generated from several ARIMA models, with innovations having different distributions denoting symmetricity or asymmetricity.
• To compare the two measures of skewness, we apply the bootstrap methodology outlined in Section ‘Bootstrap test and power comparisons’. This involves comparing the average estimates over a set number of simulations, along with comparing the powers of both the test statistics.
• It is believed that if a series is indeed symmetric, the skewness measure should hover around 0, and farther away from 0 otherwise.

## Real data analysis

**Fig. 2:**
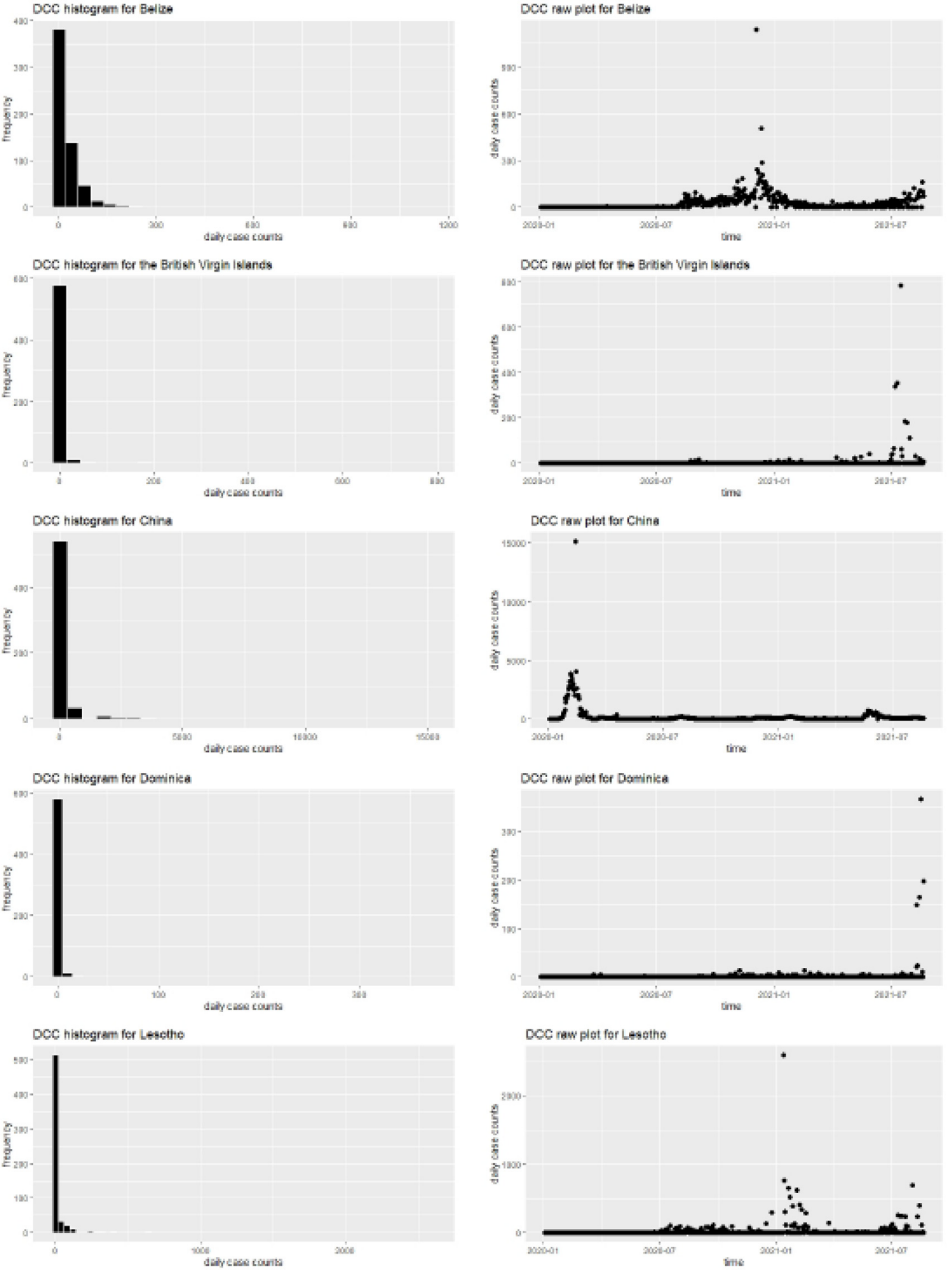
Histograms (left) and raw DCC plots (right) for the countries.

We now explore the practicality and advantages of our newly proposed skewness measure $\left(\tau_{d}\right)$ over the existing skewness measure (γ) on understanding the COVID-19 pandemic behavior towards being symmetric or highly asymmetric. Intuitively, for most countries, lower daily case counts (DCC) amount to higher frequencies. Hence such DCC will resemble an asymmetric or more specifically a right-skewed pattern. In this analysis, we will only focus on the DCC for a group of selected countries across the world. The countries in this group are Belize, British Virgin Islands, China, Dominica and Lesotho, where their DCC are seen to be highly right skewed (asymmetric) with a skewness value of atleast 10. We look at data from January 3, 2020 to August 20, 2021. Fig. 2 showcases the respective histograms and raw DCC plots for each of these countries. From these, interestingly, one can note that in British Virgin Islands and Dominica, the surge in DCC happened much later in time as compared to other countries. Of course the primary reason behind this being that both these countries are island nations situated in the Caribbean which might have restricted the spread of the virus. Also, all the histograms clearly show a tendency of being asymmetric. Further for convenience, Table 3 contains some descriptive summary statistics for DCC of the above countries.

**Table 3:** Summary statistics for the daily case counts.

Country	Mean	Minimum	Maximum	$Q_{1}$	Median	$Q_{3}$
Belize	25.47	0	1141	0	3.5	34
British Virgin Islands	4.309	0	784	0	0	0
China	205.7	0	15152	23	47	113
Dominica	1.929	0	367	0	0	0
Lesotho	24.11	0	2593	0	0	2

Now before we compare the asymmetry through the different skewness measures, we fit a suitable time series model to the DCC of these countries. The auto.arima function in the forecast package of R was used to obtain the best fit. For a comparison, Table 4 outlines these results. Again interestingly, all the models exhibit an integration of order 1.

**Table 4:** Suitable model fits to the daily case counts.

Country	Model	AR Parameters	MA Parameters
Belize	ARIMA(1,1,3)	(−0.8)	(−0.04,−0.73,0.08)
British Virgin Islands	ARIMA(1,1,5)	(−0.2)	(−0.9,0.28,−0.33,−0.33,0.73)
China	ARIMA(0,1,1)	-	(−0.7)
Dominica	ARIMA(4,1,4)	(−0.7,−0.45,−0.51,−0.62)	(−0.43,−0.1,−0.45,0.88)
Lesotho	ARIMA(2,1,3)	(−0.56,−0.87)	(−0.28,0.31,−0.87)

In order to compare the powers of the two skewness quantities, we again adopt the bootstrap technique mentioned in Section ‘Simulations and comparisons’, and a brief outline is as follows: for a particular country, we look at its DCC and assuming that it is symmetric around its mean, construct 2T linear transformations as given in Step 2 above. We further execute Steps 3 and 4. Lastly to find out the power, we consider the observed DCC series. One should note that in this case, since there is a single iteration of the observed series, the power could be either 0 or 1. Table 5 contains the skewness summaries along with the corresponding powers for each country. Now since all the DCC are integrated with order 1, we only compare γ and $\tau_{1}$. As one can note, for all the countries, the newly proposed skewness measure is capable of capturing the asymmetry perfectly and proves to be better than the existing one in terms of power.

**Table 5:** Power comparisons pertaining to the real data.

Country	γ	$P_{1}$	$\tau_{1}$	$P_{2}$
Belize	10.89	0	4.41	1
British Virgin Islands	15.17	0	1.07	1
China	12.50	0	4.07	1
Dominica	14.76	0	0.84	1
Lesotho	14.25	0	4.34	1

## Concluding thoughts

In this paper we proposed a new measure of skewness for time series which are integrated by a certain order. This proposed measure was seen to be performing better than the classical measure of skewness. A bootstrap focused power comparison was undertaken in this regard. The applicability of our proposed measure was seen through implementing it on the daily COVID case counts of a group of selected countries across the globe.

## Ethics statements

NA

## CRediT authorship contribution statement

**Sudeep R. Bapat:** Conceptualization, Investigation, Writing – original draft.

## Declaration of Competing Interest

The authors declare that they have no known competing financial interests or personal relationships that could have appeared to influence the work reported in this paper.

## Data Availability

The datasets analysed during the current study are publicly available in the following repository: https://data.humdata.org/dataset/novel-coronavirus-2019-ncov-cases
